# Peripheral blood cellular profile at pre-lymphodepletion is associated with CD19-targeted CAR-T cell-associated neurotoxicity

**DOI:** 10.3389/fimmu.2022.1058126

**Published:** 2023-01-16

**Authors:** Serena De Matteis, Michele Dicataldo, Beatrice Casadei, Gianluca Storci, Noemi Laprovitera, Mario Arpinati, Enrico Maffini, Pietro Cortelli, Maria Guarino, Francesca Vaglio, Maria Naddeo, Barbara Sinigaglia, Luca Zazzeroni, Serafina Guadagnuolo, Enrica Tomassini, Salvatore Nicola Bertuccio, Daria Messelodi, Manuela Ferracin, Massimiliano Bonafè, Pier Luigi Zinzani, Francesca Bonifazi

**Affiliations:** ^1^ IRCCS Azienda Ospedaliero-Universitaria di Bologna, Bologna, Italy; ^2^ Department of Experimental, Diagnostic and Specialty Medicine (DIMES), University of Bologna, Bologna, Italy; ^3^ Dipartimento di Scienze Biomediche e Neuromotorie, Università di Bologna, Bologna, Italy; ^4^ IRCCS Istituto delle Scienze Neurologiche di Bologna, Bologna, Italy; ^5^ Department of Medical and Surgical Sciences (DIMEC), University of Bologna, Bologna, Italy

**Keywords:** chimeric antigen receptor, senescence, inflammation, neurotoxicity, myeloid activation

## Abstract

**Background:**

Infusion of second generation autologous CD19-targeted chimeric antigen receptor (CAR) T cells in patients with R/R relapsed/refractory B-cell lymphoma (BCL) is affected by inflammatory complications, such as Immune Effector Cell-Associated Neurotoxicity Syndrome (ICANS). Current literature suggests that the immune profile prior to CAR-T infusion modifies the chance to develop ICANS.

**Methods:**

This is a monocenter prospective study on 53 patients receiving approved CAR T-cell products (29 axi-cel, 24 tisa-cel) for R/R-BCL. Clinical, biochemical, and hematological variables were analyzed at the time of pre-lymphodepletion (pre-LD). In a subset of 21 patients whose fresh peripheral blood sample was available, we performed cytofluorimetric analysis of leukocytes and extracellular vesicles (EVs). Moreover, we assessed a panel of soluble plasma biomarkers (IL-6/IL-10/GDF-15/IL-15/CXCL9/NfL) and microRNAs (miR-146a-5p, miR-21-5p, miR-126-3p, miR-150-5p) which are associated with senescence and inflammation.

**Results:**

Multivariate analysis at the pre-LD time-point in the entire cohort (n=53) showed that a lower percentage of CD3^+^CD8^+^ lymphocytes (38.6**%** vs 46.8%, OR=0.937 [95% CI: 0.882-0.996], p=0.035) and higher levels of serum C-reactive protein (CRP, 4.52 mg/dl vs 1.00 mg/dl, OR=7.133 [95% CI: 1.796-28], p=0.005) are associated with ICANS. In the pre-LD samples of 21 patients, a significant increase in the percentage of CD8^+^CD45RA^+^CD57^+^ senescent cells (median % value: 16.50% vs 9.10%, p=0.009) and monocytic-myeloid derived suppressor cells (M-MDSC, median % value: 4.4 vs 1.8, p=0.020) was found in ICANS patients. These latter also showed increased levels of EVs carrying CD14^+^ and CD45^+^ myeloid markers, of the myeloid chemokine CXCL-9, as well of the MDSC-secreted cytokine IL-10. Notably, the serum levels of circulating neurofilament light chain, a marker of neuroaxonal injury, were positively correlated with the levels of senescent CD8^+^ T cells, M-MDSC, IL-10 and CXCL-9. No variation in the levels of the selected miRNAs was observed between ICANS and no-ICANS patients.

**Discussion:**

Our data support the notion that pre-CAR-T systemic inflammation is associated with ICANS. Higher proportion of senescence CD8^+^ T cells and M-MDSC correlate with early signs of neuroaxonal injury at pre-LD time-point, suggesting that ICANS may be the final event of a process that begins before CAR-T infusion, consequence to patient clinical history.

## 1 Introduction

A large number of chimeric constructs have been developed and tested so far, but only a few second-generation CAR-T cell products (e.g., anti-CD19/CD137 chimeric and CD19/CD28 chimeric) are currently used in clinical practice (1, 2). The overall response rate to CAR-T cells in lymphoma patients is approximately 50-60% (3, 4) and its efficacy is hampered by systemic hyper-inflammatory adverse events, including cytokines release syndrome (CRS) and Immune effector Cell-Associated Neurotoxicity Syndrome (ICANS). The clinical manifestations of ICANS are heterogeneous, ranging from language disturbances and frontal-predominant encephalopathy to akinetic mutism and, anecdotally, fulminant diffuse cerebral oedema (5–9). Patients with high baseline inflammatory serum profile as defined by pro-inflammatory cytokines (e.g., IL-6), C-reactive protein (CRP), ferritin and D-dimer have increased risk of developing ICANS (10–12).

Although neurotoxicity is associated with elevation of pro-inflammatory cytokines, the pathophysiology of ICANS is quite unclear, thus limiting its appropriate clinical management, mostly based on corticosteroids administration (13). Literature data show that the immune myeloid compartment plays a central role in ICANS onset (5). In particular, monocyte-derived inflammatory cytokines, such as IL-6, can increase the permeability of the blood brain barrier (BBB) and promote myeloid cell infiltration and neurotoxicity (13–16).

Notably, CAR-T cell-induced neurotoxicity can be largely influenced by the pre-treatment tumor immune microenvironment, and by the crosstalk among lymphoma cells, myeloid cells, cytotoxic and regulatory T cells (17).

In this monocenter prospective study, we aimed at investigating pre-LD peripheral blood cellular and biochemical features in patients infused with CAR-T cell therapy to identify potentially predictive markers of the pathologic processes that will ultimately lead to ICANS onset.

## 2 Materials and methods

### 2.1 Patients’ enrollment

Patients with diffuse large B‐cell lymphoma (DLBCL), DLBCL from transformed follicular or marginal zone lymphoma (tFL, tMZL), high grade (HGBCL) and primary mediastinal B‐cell lymphoma (PMBCL) were enrolled in a prospective monocenter observational tissue study. The decision to use either axi‐cel or tisa‐cel was based on slot production availability and histology, according to each product approval. All patients received lymphodepleting chemotherapy with fludarabine and cyclophosphamide from day ‐5 to day ‐3 (fludarabine: 25–30 mg/m2 and cyclophosphamide: 250–500 mg/m2). Patients were hospitalized from lymphodepletion to at least 14 days after CAR-T cells infusion in an HEPA-filtered room, according to the institutional SOPs. CRS and ICANS were graded according to the American Society for Transplantation and Cellular Therapy (ASCT) criteria (6). The study was conducted according to the Helsinki declaration and approved by the local Ethical Committee. The study was registered at clinicalTrials.gov (NCT04892433). All patients signed a written informed consent.

### 2.2 Samples collection

Peripheral blood samples were collected from each patient at pre-apheresis (pre-AP), pre-lymphodepletion (pre-LD) and at +3, +7, +13, +21, +30, +90, +180 days after CAR-T cell infusion, when available.

### 2.3 Flow cytometry analysis

Flow cytometric analysis was performed using a 3-laser FacsCanto II (BD Biosciences, San Jose, CA). A minimum of 50.000 CD45^+^ lymphocytes has been recorded for each analysis. Flow cytometry data were analyzed with DiVa 6.1.1 software and FCS Express 7 Reader. Appropriate isotype controls were included for each sample.

CAR-T cell tracking was performed on fresh whole blood at all available time-points and on 100 μl of bag leftovers (18). Cells were stained with the CD19 CAR FMC63 Idiotype antibody-APC (Miltenyi Biotec, Bergisch-Gladbach, Germany) following the manufacturer’s instruction. Then, cells were labeled with the following set of antibodies: CD45, CD3, CD4, CD8, CD45RA, CD62L, CD57, CD28 (from BD Biosciences). The analysis of monocytic (M-) and polymorphonuclear (PMN-) myeloid derived suppressor cells (MDSC) was performed on peripheral blood mononuclear cells (PBMC) separated by density gradient centrifugation using Lymphosep (Biowest) within 4 h after pre-LD sample collection. The following mAbs were used for MDSC identification: CD11b, CD14, CD15, CD33, CD45, and HLA-DR (from BD Biosciences). A minimum of 100.000 events has been recorded in the PBMC-gated population.

### 2.4 ELISA test

Plasma levels of cytokines (IL-6, IL-10, IL-15, GDF-15), chemokine CXCL-9 and neuronal damage marker Neurofilament light chain (NfL) were assessed by high performance multi analyte microfluidic system Protein ELLA (19) on pre-LD samples (Biotechne, San Josè California).

### 2.5 CAR-T molecular tracking by droplet digital PCR

DNA was isolated from 200 µl of stored whole blood at each timepoint using QIAamp DNA Mini and Blood Mini kit (Qiagen, Germany) eluting in 50 µl of Buffer AE. DNA yield and quality were assessed with NanoGenius Spectrophotometer (ONDA Spectrophotometer, Giorgio Bormac s.r.l., Carpi, Italy). The molecular tracking of the transgenic CAR was performed by probe-based Droplet Digital PCR detecting and quantifying CAR T DNA (absolute copies) using constant volumes of DNA input (range of 20-80 ng) and Axi-Cel/Tisa-Cel Universal CD19-CAR T DNA assay (Bio-Rad, USA).

### 2.6 Circulating microRNA quantification

For each patient at pre-LD, RNA was purified from 200 µl of plasma collected at pre-LD using Maxwell RSC miRNA Plasma/Serum Kit and Maxwell RSC Instrument (Promega, USA). miRNA polyadenylation and reverse transcription were performed using miRCURY LNA RT kit (Qiagen, Germany). A panel of SIP-associated circulating microRNAs including miR-146a-5p, miR-21-5p, miR-126-3p, miR-150-5p, has been assessed in each sample by EvaGreen-based Droplet Digital PCR (Bio-Rad, USA) and miRCURY LNA primers (Qiagen, Germany). Positive droplet selection was performed using QuantaSoft software (v. 1.7) to obtain the final absolute levels of each miRNA (expressed in copies/µl).

### 2.7 Exosomes phenotypic characterization

Plasma samples collected at pre-LD were subjected to bead-based multiplex EV analysis by flow cytometry (MACSPlex Exosome Kit, Miltenyi Biotec) according to the manufacturer’s instructions and as previously reported (20).

### 2.8 Statistical analysis

In the whole cohort of 53 treated patients, descriptive statistics are reported as mean and standard deviation for continuous variables and as percentage for categorical ones. The statistical association of clinical and laboratory variables with ICANS were assessed in the entire population through Fisher exact test, Mann-Withney U-test and one-way ANOVA, depending on categorical, non-normal and normal distribution, respectively. Log transformation of non-normal laboratory variables allowed to obtain a parametric distribution. Two-tail significance cut-off of 0.05 was chosen and all variables significantly associated with ICANS were evaluated in a multivariate logistic regression. Correlation among variables was measured by means of Pearson’s coefficient. All statistical analyses were performed with SPSS 22.0 (IBM Corp. Armonk, NY).

## 3 Results

A total of 53 patients (47 DLBCL, 6 PMBCL) underwent leukapheresis and CAR-T cell infusion at our Institution. Specifically, 29 patients received axi‐cel and 24 received tisa‐cel CAR-T cell product. Baseline characteristics of treated patients are reported in **Table 1**. The median age was 57 years (range 19-70), 38 patients were in progressive disease and 43 received bridging therapy before infusion. A total of 45 patients developed CRS of any grade (**Table 2**): 26 cases grade 1; 19 grade ≥ 2 and 5 grade 3-4. Eighteen patients developed ICANS of any grade (**Table 2**): 11 patients had grade 1-2 (6 treated with axi-cel and 5 treated with tisa-cel), 7 patients had grade 3-4 (all patients treated with axi-cel). The median time of CRS onset was 2 days after infusion (range 0-11 days), with 13 cases occurring the same day of infusion, whereas the time of ICANS onset was 5 days after infusion (range 0-12 days).

**Table 1:** Patients’ characteristics.

MEDIAN AGE (range)	57 (19-70)
**GENDER** Male Female	37 (69.8%) 16 (30.2%)
**ECOG** 0-1 2-3	48 (90.6%) 5 (9.4%)
**HCT-CI** 0-1 2-3 4-6	22 (41.5%) 23 (43.4%) 8 (15.1%)
**DIAGNOSIS** DLBCL, NOS t-DLBCL HGBCL PMBCL	27 (50.9%) 17 (32.1%) 3 (5.7%) 6 (11.3%)
**STAGING** NA I-II III-VI	1 (1.9%) 10 (18.9%) 42 (79.2%)
**IPI SCORE** NA 0-1 2-4	27 (50.9%) 7 (13.2%) 19 (35.9%)
**DISEASE STATUS** PD SD/PR	38 (71.7%) 15 (28.3%)
**MEDIAN LINES OF THERAPY (range)**	3 (2-11)
**ASCT** YES NO	14 (26.4%) 39 (73.6%)
**BRIDGING THERAPY** YES NO	43 (81.1%) 10 (18.9%)
**CELLULAR INFUSED PRODUCT** Axi-cel Tisa-cel	29 (54.7%) 24 (45.3%)
**TOTAL**	53

ECOG, Eastern Cooperative Oncology Group; HCTI-CI, Hematopoietic cell transplantation-specific comorbidity index; DLBCL, NOS, Diffuse Large B-Cell Lymphoma, not otherwise specified; t-FL, transformed Follicular Lymphoma; FL, Follicular Lymphoma; GZL, “Gray-zone” Lymphoma; HGBCL, High grade b cell lymphoma; PMBCL, Primary mediastinal large b cell lymphoma; PD, Progressive disease; SD, Stable disease; PR, partial response; IPI, International Prognostic Index; ASCT, Autologous stem cell transplant.

**Table 2:** Clinical complications after CAR-T infusion.

		TISA-CEL	AXI-CEL
**CRS** YES NO	45 (84.9%) 8 15.1%)	20 (83.3%) 4 (16.7%)	25 (86.2%) 4 (13.8%)
**MEDIAN ONSET (days)**	2 (0-11)	1 (0-7)	2 (0-11)
**GRADE OF CRS** **1** **2** **3** **4**	26 (57.8%%) 14 (31.1%) 3 (6.7%) 2 (4.4%)	12 (60%) 7 (35%) 0 1 (5%)	14 (56%) 7 (28%) 3 (12%) 1 (4%)
**MEDIAN DOSES OF TOCILIZUMAB**	3 (1-4)	3 (1-3)	3(1-4)
**INCANS** YES NO	18 (34%) 35 (66%)	5 (20.8%) 19 (79.2%)	13 (44.8%) 16 (55.2%)
**MEDIAN ONSET (days)**	5 (0-12)	4 (1-12)	5 (0-11)
**GRADE OF ICANS** **1** **2** **3** 4*	5 (27.8%) 6 (33.3%) 3 (16.7%) 4 (22.2%)	3 (60%) 2 (40%) 0 0	2 (15.4%) 4 (30.7%) 3 (23.2%) 4 (30.7%)
**LINES OF THERAPY** TOCILIZUMAB 8 mg/kg 6-METHYLPREDNISOLONE 1 mg/kg q12h DEXAMETHASONE 10 mg q6h DEXAMETHASONE 20 mg q6h 6-METHYLPREDNISOLONE 1000 mg QD ANAKINRA 100 mg q12h SILTUXIMAB 11mg/kg	32 (46.4%) 13 (18.8%) 12 (17.4%) 1 (1.4%) 5 (7.2%) 3 (4.4%) 3 (4.4%)	14 (60.9%) 5 (21.7%) 3 (13.1) 0 1 (4.3%) 0 0	18 (39.1%) 8 (17.4%) 9 (19.6%) 1 (2.2%) 4 (8.7%) 3 (6.5%) 3 (6.5%)
**ICU TRANSFERRAL** YES NO	11 (20.8%) 42 (79.2%)	3 (12.5%) 21 (87.5%)	8 (27.6%) 21 (72.4%)
**INVASIVE VENTILATION**	3 (5.7%)	0	3 (5.7%)
**MEDIAN DURATION FROM ICU ADMISSION (days)**	9 (1-25)	4 (1-25)	10 (2-18)

ICANS, Immune effector cell-associated neurotoxicity syndrome; CRS, Cytokine release syndrome; ICU, Intensive care unit.

*2 people died for neurological complications, 2 and 11 days after neurotoxicity onset respectively.

In the overall cohort of 53 patients, the univariate analysis conveyed statistically significant associations between lower total and CD8+ lymphocytes, INR, Fibrinogen, CRP and ICANS (**Table 3**). Multivariate analysis confirmed that lower level of CD3^+^CD8^+^ lymphocytes (38.61**%** vs 46.82%, OR=0.937 [95% CI: 0.882-0.996], p=0.035) and higher CRP serum level (4.52 mg/dl vs 1.00 mg/dl, OR=7.133 [95% CI: 1.796-28.133], p=0.005) are independently associated to ICANS (**Table 4**).

**Table 3:** Univariate analysis of ICANS.

	ICANS		Sig.
Mean (st. dev.)	YES	NO	
Age	51.2 (17.6) yrs	55.6 (12.3) yrs	0.298
Hemoglobin	10.97 (1.38) g/dL	11.54 (1.79) g/dL	0.142
Platelets	171 (97) x 10^9/L	193 (74) x 10^9/L	0.354
White blood cells	5.306 (3.207) x 10^9/L	5.677 (2.751) x 10^9/L	0.672
Neutrophils	3.859 (2.689) x 10^9/L	3.877 (2.456) x10^9/L	0.961
Lymphocytes	0.731 (0.559) x 10^9/L	1.117 (0.700) x 10^9/L	**0.021**
Monocytes	0.469 (0.625) x10^9/L	0.525 (0.262) x10^9/L	0.731
Eosinophils	0.064 (0.069) x10^9/L	0.128 (0.256) x10^9/L	0.352
Basophils	0.026 (0.029) x10^9/L	0.030 (0.023) x10^9/L	0.730
CD3^+^	81.5 (12.9) %	84.4 (10.6) %	0.382
CD3^+^CD4^+^	41.7 (18.0) %	35.4 (14.1) %	0.170
CD3^+^CD8^+^	38.5 (13.8) %	46.9 (14.1) %	**0.045**
CD4^+^/CD8^+^	1.7 (2.7)	0.9 (0.6)	0.108
CD56^+^CD16^+^CD3^-^	11.6 (5.5) %	13.7 (10.3) %	0.508
CD19^+^	6.2 (10.2) %	1.3 (4.1) %	**0.018**
INR	0.97 (0.25)	0.96 (0.18)	**0.017**
aPTT	0.85 (0.16)	0.84 (0.17)	0.404
Fibrinogen	445 (131) mg/dL	342 (109) mg/dL	**0.004**
D-Dimer	1.31 (1.86) mcg/mL	0.69 (0.84) mcg/mL	0.178
Triglycerides	156 (106) mg/dL	169 (70) mg/dL	0.594
LDH	382 (262) U/L	313 (265) U/L	0.372
Ferritin	827 (1637) ng/mL	322 (332) ng/mL	0.083
LogFerritin	2.35 (0.77)	2.29 (0.48)	0.746
CRP	4.52 (6.42) mg/dL	1.03 (1.76) mg/dL	**0.005**
LogCRP	0.18 (0.70)	-0.37 (0.57)	**0.002**
IL-6	17.1 (22.4) pg/mL	8.2 (9.2) pg/mL	0.077
LogIL-6	0.98 (0.51)	0.75 (0.38)	0.089
IgG	481 (195) mg/L	572 (251) mg/L	0.209
**DISEASE STATUS** PD PR SD	15 (28.3%) 1 (1.9%) 2 (3.8%)	23 (43.4%) 8 (15.1%) 4 (7.5%)	0.271
**Gender** Female Male	9 (17%) 9 (17%)	7 (13.2%) 28 (52.8%)	**0.032**
**HCT-CI** 0-1 ≥2	5 (9.4%) 13 (24.5%)	17 (32.1%) 18 (34%)	0.239
**CELLULAR PRODUCT** Axi-cel Tisa-cel	13 (24.5%) 5 (9.4%)	16 (30.2%) 19 (35.9%)	0.085
**BRIDGING THERAPY** No Yes	1 (1.9%) 17 (32.1%)	9 (17%) 26 (49%)	0.137
**LINES OF THERAPY** 2-3 ≥4	11 (20.8%) 7 (13.2%)	23 (43.4%) 12 (22.6%)	0.770
**DIAGNOSIS** PMBCL Other	5 (9.4%) 13 (24.5%)	1 (1.9%) 34 (64.2%)	**0.014**

ICANS, Immune effector cell-associated neurotoxicity syndrome; CD3+, T-lymphocytes; CD3+CD4+, CD4+ subtype of T-lymphocytes; CD3+CD8+, CD8+ subtype of T-lymphocytes; CD4+/CD8+, ratio between two subtypes; CD3-CD56+CD16+, NK-cells; CD19+, B-lymphocytes; INR, international normalized ratio; aPTT, activated partial thromboplastin clotting time; CRP, C reactive protein; LogCRP, log transformation of CRP; PMBCL, Primary mediastinal large b cell lymphoma; PD, Progressive disease; SD, Stable disease; PR, partial response; HCTI-CI, Hematopoietic cell transplantation-specific comorbidity index.The bold characters indicate the statistically significant values.

**Table 4:** Multivariate analysis of ICANS.

	OR (95% CI)	Sig.
CRP	7.133 (1.796-28.323)	**0.005**
% CD3^+^CD8^+^ T cells in peripheral blood	0.937 (0.882-0.996)	**0.035**
DIAGNOSIS (PMLBCL vs Other)	19.454 (1.490-254.007)	**0.024**

LogPCR, log transformation of CRP; CD3+CD8+, CD8+ subtype of T-lymphocytes; PMBCL, Primary mediastinal large b cell lymphoma.The bold characters indicate the statistically significant values.

**Figure 1:**
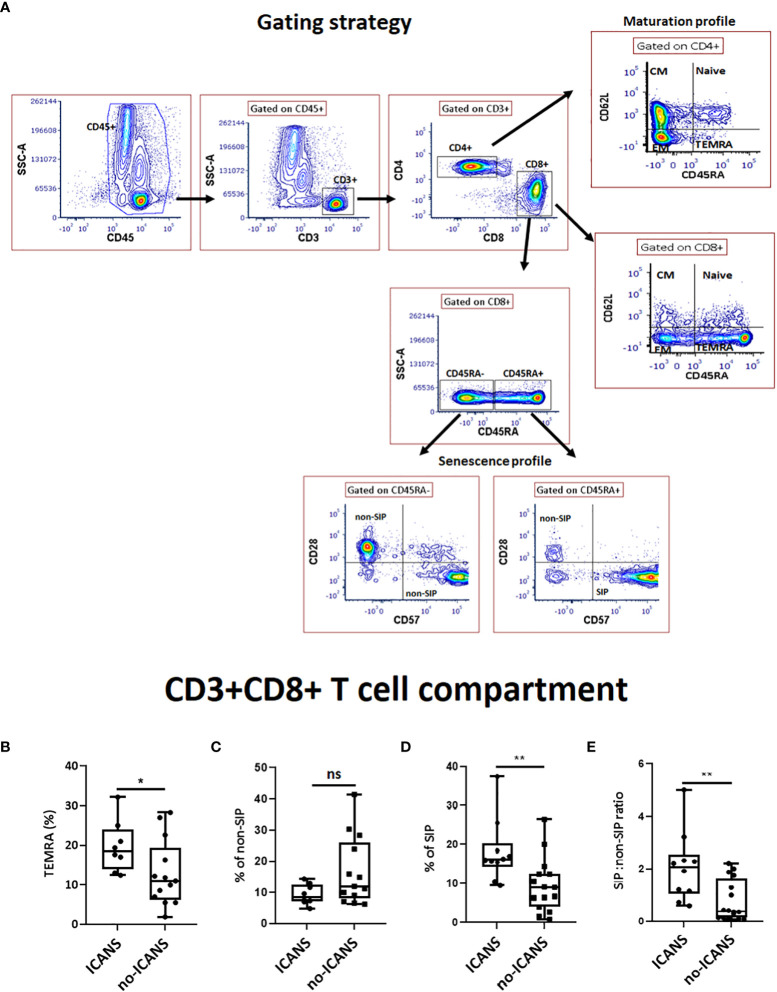
Cytofluorimetric analysis. **(A)** Gating strategy used to identify CD8^+^ T cell subsets. A lymphocyte gate was set based on the CD45^+^ and SSC parameters. Among CD3^+^CD4^+^ or CD3^+^CD8^+^ compartments, different subsets were analyzed: Naive (CD45RA^+^62L^-^), central memory (CM) (CD45RA^-^62L^+^), effector memory (EM) (CD45RA^-^62L^-^), terminally differentiated effector (TEMRA) (CD45RA^+^62L^-^) T cells; Among CD3^+^CD8^+^ compartment, SIP^+^ cells were measured as percentage of CD45RA^+^28^-^57^+^ whereas CD45RA^+^28^+^, CD45RA^-^57^+^ and CD45RA^-^28^+^ among CD8^+^ T cells were defined as non-SIP. The box plots show the changes in the percentage of **(B)** TEMRA, **(C)** non-SIP, **(D)** SIP, **(E)** SIP:non-SIP ratio among CD3^+^CD8^+^ T cell compartment in ICANS and no-ICANS patients. Comparisons between 2 groups were made using the non-parametric, unpaired Mann-Whitney test. *p <0.05; **p <0.01; ns = not significant.

Prompted by these results, peripheral blood leukocytes collected at pre-LD time-point were analyzed by cytofluorimetric analysis in a subgroup of 21 patients whose fresh blood sample was available. The gating strategy was specifically designed to address the presence of CD45RA^+^CD62L^-^ terminally differentiated effector (TEMRA) cells, CD45RA^+^CD28^-^CD57^+^ senescent immune phenotype (SIP) cells and non-SIP cells defined as CD45RA^+^CD28^+^CD57^-^, CD45RA^-^CD28^+^CD57^-^, CD45RA^-^CD28^-^CD57^+^ in the CD8+ compartment (**Figure 1A**). A higher percentage of CD8^+^TEMRA (median value: 18.50 vs 11.00, p=0.030), CD8^+^SIP cells (median value: 16.50 vs 9.10, p=0.009), as well as the SIP over non-SIP ratio (2.24 vs 0.42, p=0.0045) were found in patients who developed ICANS (**Figures 1B–E**). No significant differences were observed when the other T cell compartments were examined (**Supplemental Figures 1A, B**). Cytofluorimetric analysis was also performed on blood leukocytes collected at the time of leuko-apheresis: a substantial interindividual heterogeneity, but no significant difference in the percentage of SIP cells and in the SIP over non-SIP ratio in patients who developed ICANS was observed (**Supplemental Figures 2A–E**). The infusion product analysis did not reveal any significant changes in T subpopulations of patients who developed ICANS (**Supplemental Figure 3A**).

Noteworthy, CAR-T cell expansion kinetics assessed by FACS analysis and ddPCR showed no statistically significant differences in the expansion peak between ICANS and no-ICANS patients. Notably, the expansion peak occurred at day 7 to day 13 post CAR-T infusion, i.e., after the median time of ICANS onset (5 days after infusion, **Supplemental Figures 3B–D**).

**Figure 2:**
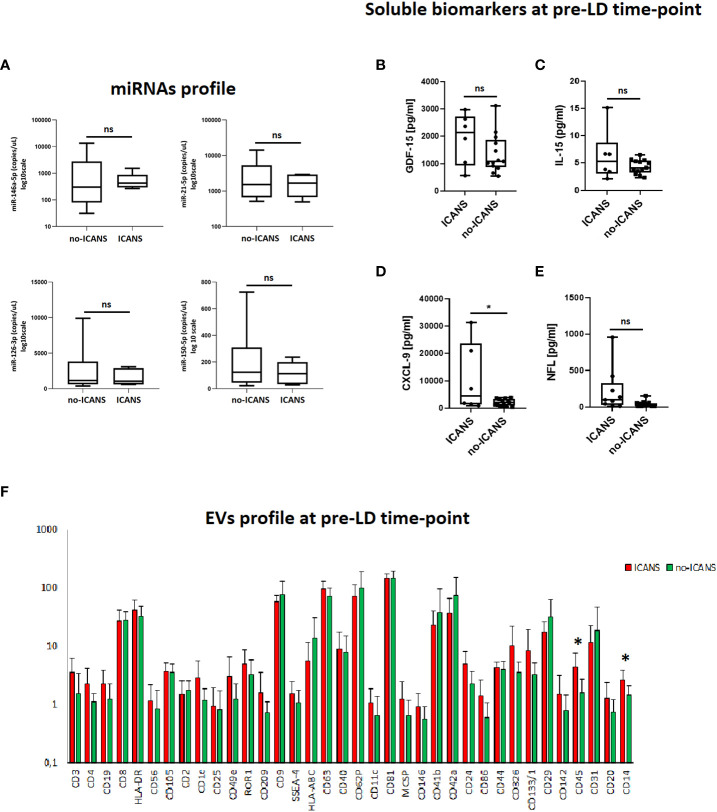
Plasma markers analysis. **(A)** Box plots report the expression levels of miR-146a-5p, miR-21-5p, miR-126-3p, miR-150-5p at pre-LD in ICANS and no-ICANS patients. Plasma levels of **(B)** GDF-15, **(C)** IL-15, **(D)** CXCL-9 and **(E)** NfL at pre-LD in ICANS and no-ICANS patients. **(F)** Range from min to max of the Mean Fluorescence Intensity (MFI) for each plasma EVs marker. Plasma from patients experiencing ICANS in red and no-ICANS patients in green; values have been normalized to blank control. Comparisons between 2 groups were made using *t* test. *p = <0.05; ns = not significant.

In the attempt to identify soluble markers of the pro-inflammatory/senescent profile at the pre-LD time-point, we assessed the plasma levels of miRNAs associated with inflammation and senescence, namely miR-146a-5p, miR-21-5p, endothelial homeostasis miR-126-3p and B-cell tumor burden miR-150-5p: no significant differences between the two groups of patients were observed (**Figure 2A**). We also assessed the pre-LD plasma levels of senescence-associated cytokines GDF-15 and IL-15, of the myeloid chemokine CXCL-9, as well as of the neuroaxonal damage marker (21) NfL (**Figures 2B–E**). The plasma level of CXCL-9 was higher in patients who experienced ICANS (mean value [pg/ml]: 10605.50 ± 12639 vs 2140.46 ± 1231.38, p=0.024). Moreover, plasma level of circulating NfL, although did not significantly differ between ICANS and no-ICANS patients (mean value [pg/ml]: 116.6 ± 151.8 vs 45.65 ± 25.77, p=0.210) were positively correlated with SIP non-SIP ratio (r=0.555, p=0.014), CXCL-9 (r=0.812, p=0.00024), IL-10 (r=0.729, p=0.001).

Interestingly, the flow cytometric analysis of EVs phenotype at pre-LD time-point revealed an enrichment of leukocyte-derived CD45^+^ EV (mean fluorescence intensity 4.37 ± 3.34 vs 1.62 ± 1.11, p=0.029) and myeloid cell-derived CD14^+^ EV (mean fluorescence intensity 2.59 ± 1.34 vs 1.48 ± 0.65, p=0.042) in patients who developed ICANS (**Figure 2F**). Notably, CD45^+^EVs were associated with the level of NfL (r=0.651, p=0.006).

**Figure 3:**
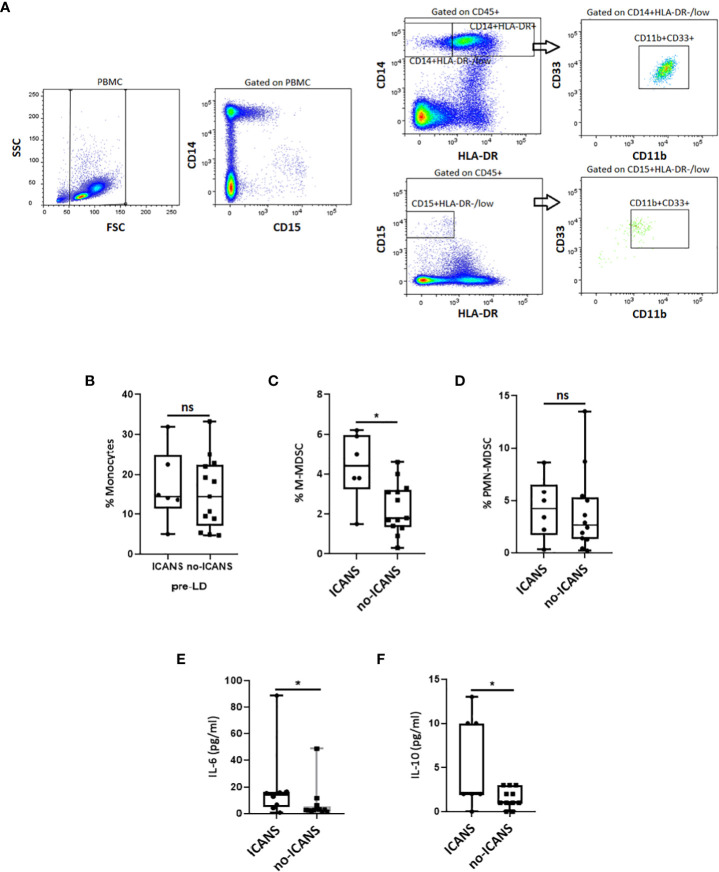
Cytofluorimetric analysis. **(A)** Gating strategy used to identify the M- and PMN-MDSC in a patient before receiving CAR-T cell infusion. The CD14^+^HLA-DR^−/low^ cell subset for M-MDSC or CD15^+^HLA-DR^-/low^ for PMN-MDSC was gated, and the proportion of CD11b^+^CD33^+^ was evaluated. The box plots report the percentage of **(B)** Monocyres, **(C)** M-MDSC, **(D)** PMN-MDSC, and plasma levels of **(E)** IL-6, **(F)** IL-10 at pre-LD in ICANS and no-ICANS patients. Comparisons between 2 groups were made using the non-parametric, unpaired Mann-Whitney test. *p <0.05; ns = not significant.

We thus evaluated the myeloid cell compartment, by assessing M-MDSC and PMN-MDSC cell subpopulations (**Figure 3A**). No difference in the percentage of monocytes (defined as CD14^+^HLA-DR^+^) (median value: 14.4 vs 14.3, p=0.687) was observed between ICANS and no-ICANS patients (**Figure 3B**). Higher percentage of M-MDSC (defined as CD14^+^HLA-DR^-/low^CD11b^+^CD33^+^) (median value: 4.40 vs 1.80, p=0.020), but not PMN-MDSC (defined as CD15^+^HLA-DR^-/low^CD11b^+^CD33^+^) (median value: 4.20 vs 2.60, p=0.578) were found in patients who developed ICANS (**Figures 3C, D**). The MDSC-inducing cytokine IL-6 (mean value [pg/ml]: 14.12 ± 2.39 vs 3.71 ± 2.69, p=0.030) and the MDSC-secreted cytokine IL-10 (mean value [pg/ml]: 5.12 ± 4.99 vs 1.45 ± 1.21, p=0.030) were increased at the pre-LD time-point in ICANS patients (**Figures 3E, F**).

## 4 Discussion

These findings support that systemic inflammation preceding CAR-T cell infusion, marked by high CRP serum levels, has a negative prognostic value for CAR-T cell toxicity (22, 23). Several markers of systemic inflammatory activation, including ferritin, IL-6 and fibrinogen (here significantly associated with ICANS in univariate analysis) have been linked to CAR-T cell toxicity (22, 24). In our series, ICANS occurred in patients who previously developed CRS. Although these two complications may occur in the same patients, the mechanisms underlying these adverse inflammatory events are likely to be different. CRS pathogenesis has been extensively studied and appropriate treatment (IL-6 axis targeting antibodies) are currently available (25–27). Instead, the pathophysiology of ICANS and its management have been less clearly defined. Higher levels of endothelial stress (e.g. angiopoietin-2) and coagulopathy (e.g. high concentration of D-dimers) markers have been showed to correlate with severe neurotoxicity (14, 16, 22, 27, 28). Notably, IL-6 receptor system blockade by tocilizumab seems to be ineffective in ICANS (29), whereas the inhibition of myeloid-derived mediators such as IL-1 is potentially capable to ameliorate neurotoxicity in the murine model (13–15) and may be of clinical relevance (30). Several sources of systemic inflammation can be envisaged in patients undergoing CAR-T cell therapy. Here, in our case series, we found that patients developing ICANS have decreased levels of CD3^+^CD8^+^ T cells and total lymphocytes at pre-LD time-point. This decrease may be the consequence of immune-senescence (31). Accordingly, in these patients we found an increase in pre-LD senescent CD8^+^ T cells. Senescent T cell onset may be due to the prolonged attrition of tumor antigens, the long-lasting exposure to senescence-inducing factors in the tumor microenvironment, as well as the exposure to cytotoxic therapies (31). Moreover, senescent T cells are poorly responsive to specific stimuli, while retaining the ability to trigger inflammation by cytokines/chemokines (GDF-15, CXCL-9 and IL-6) secretion (32, 33). In turn, the plasma/serum levels of these mediators have been associated with frailty and poor outcome in a variety of diseases (32–34).

At least in the subgroup of 21 cases, we found higher pre-LD IL-6 serum level in patients developing ICANS. This cytokine acts as the main regulator of MDSC-driven compensatory circuitry, which physiologically switches-off inflammation (32). However, when the MDSC-driven feedback loop stalls, it creates a combination of inflammation and immunodepression that is, on the one hand, favorable for tumor progression and, on the other hand, unfavorable for an efficient immune surveillance, thus thwarting the efficacy of immunotherapy. Accordingly, we found high levels of M-MDSC in patients later developing ICANS. We found that IL-6 (hallmark of the MDSC secretome (32)) levels were positively correlated with the percentage of MDSC. Noteworthy, despite its powerful anti-inflammatory activity, higher IL-10 levels have been previously linked to neurotoxicity (5, 16). It is also worth noting that higher MDSC levels have been linked to a blunted CAR-T cell expansion (12). To reinforce the notion that MDSC up-regulation is the telltale of myeloid compartment activation at pre-LD time-point, we found high levels of the myeloid specific chemokine CXCL-9 in patients who developed ICANS. In addition, CXCL-9 levels were found to be highly correlated with CD45^+^ EVs and IL-10 plasma levels, as well as senescent CD8^+^ T cells. Notably, it has been reported that CXCL-9 expression in the tumor microenvironment at the pre-treatment stage affects the local immune landscape in patients treated with CAR-T cell therapy (17). Speculatively, CXCL-9 circulating levels may provide information about the role of the tumor microenvironment in the onset of ICANS (17). Moreover, since CXCL-9 takes part to senescent secretome (34), our data corroborate the notion that at pre-LD time-point, systemic inflammation and myeloid cells activation are predisposing conditions for CAR-T cell toxicity and may functionally linked each other. Notably, despite we could not document a clear difference between ICANS and no-ICANS patients, at the pre-LD time-point circulating NfL serum levels were positively correlated with myeloid markers (CXCL-9, IL-10, CD45^+^ EVs) and with the percentage of senescent CD8^+^ T cells. These data suggest that a latent neuronal damage is already present before CAR-T treatment, thus predisposing to the later ICANS (32). In keeping with this reasoning, high plasma levels of NfL before CAR-T infusion have been associated with severe ICANS in larger case series (35). In conclusion, our data suggest that pre-CAR-T systemic inflammation associated with the ICANS onset is linked to CD8^+^ T cells senescence, myeloid compartment activation and preexisting neuroaxonal injury.

ICANS is therefore likely to be the final event of a pathologic process that begins before the infusion of CAR-T cell therapy. Since we did not detect signs of CD8^+^ T cells senescence in patients who later developed ICANS at the pre-apheresis time-point, the phenomenon could be also associated with the bridging therapy regimen, especially if chemotherapy-based. A larger sample and/or multicenter studies are warranted to verify this hypothesis and its implication in the management of CAR-T patients.

## Data availability statement

The original contributions presented in the study are included in the article/**Supplementary Material**. Further inquiries can be directed to the corresponding author.

## Ethics statement

The studies involving human participants were reviewed and approved by Ethical Committee AVEC of Bologna. IRCCS Azienda Ospedaliero-Universitaria di Bologna, Bologna, Italy. The study was registered at clinicalTrials.gov (NCT04892433). The patients/participants provided their written informed consent to participate in this study.

## Author contributions

Contribution: SM, MD, MB, PLZ and FB initiated the project, designed the research, and wrote the paper with input from other authors; SM, GS, NL, MF, FV, MN, SG, ET, SB, DM, MB, FB performed correlative laboratory studies, assisted with data analysis; BS and LZ performed CAR T-cells thawing and processing in the tissue establishment; MD, BC, MA, EM, PC, MG, PLZ and FB provided clinical data and clinical care for the patient. All authors contributed to the article and approved the submitted version.

## Supplementary material

Supplementary Figure 1 Cytofluorimetric analysis. The box plots show the changes in the percentage of Naïve, CM, EM and TEMRA among **(A)** CD3^+^CD8^+^ and **(B)** CD3^+^CD4^+^ T cell compartments at pre-LD in ICANS and no-ICANS patients. Comparisons between 2 groups were made using the non-parametric, unpaired Mann-Whitney test. **p* = <0.05; ***p* = <0.01; ****p* = <0.001.

Supplementary Figure 2 Cytofluorimetric analysis. Scatter plots report the distribution of different maturation subsets among **(A)** CD4, **(B)** CD8, **(C)** SIP and non-SIP in CD3^+^CD8^+^ T cell compartment at pre-AP; Box plots report the changes in the percentage of SIP **(D)** and SIP:non-SIP ratio **(E)** among CD3^+^CD8^+^ T cell compartment at pre-AP **(F)**.

Supplementary Figure 3 Cytofluorimetric analysis. **(A)** Histograms show the comparison of frequency distribution of Naive, CM, EM and TEMRA cells gated on CD3^+^CAR^+^CD8^+^ cell compartment in infusion product bags leftovers stratified according ICANS. **(B)** Representative gating strategy of CAR^+^ T cell population at expansion peak (day+7). CAR-T cell expansion kinetics in patients receiving axi-cel and tisa-cel assessed by **(C)** flow cytometry and **(D)** ddPCR (red: patients who developed ICANS; black: no-ICANS patients. Comparisons between 2 groups were made using the non-parametric, unpaired Mann-Whitney test. **p* = <0.05; ***p* = <0.01; ****p* = <0.001.
