# Supplementary figures and images for: Peripheral blood cellular profile at pre-lymphodepletion is associated with CD19-targeted CAR-T cell-associated neurotoxicity
Source: Front Immunol. 2023 Jan 16;13:1058126. doi: 10.3389/fimmu.2022.1058126 (PMC9886226; doi:10.3389/fimmu.2022.1058126)

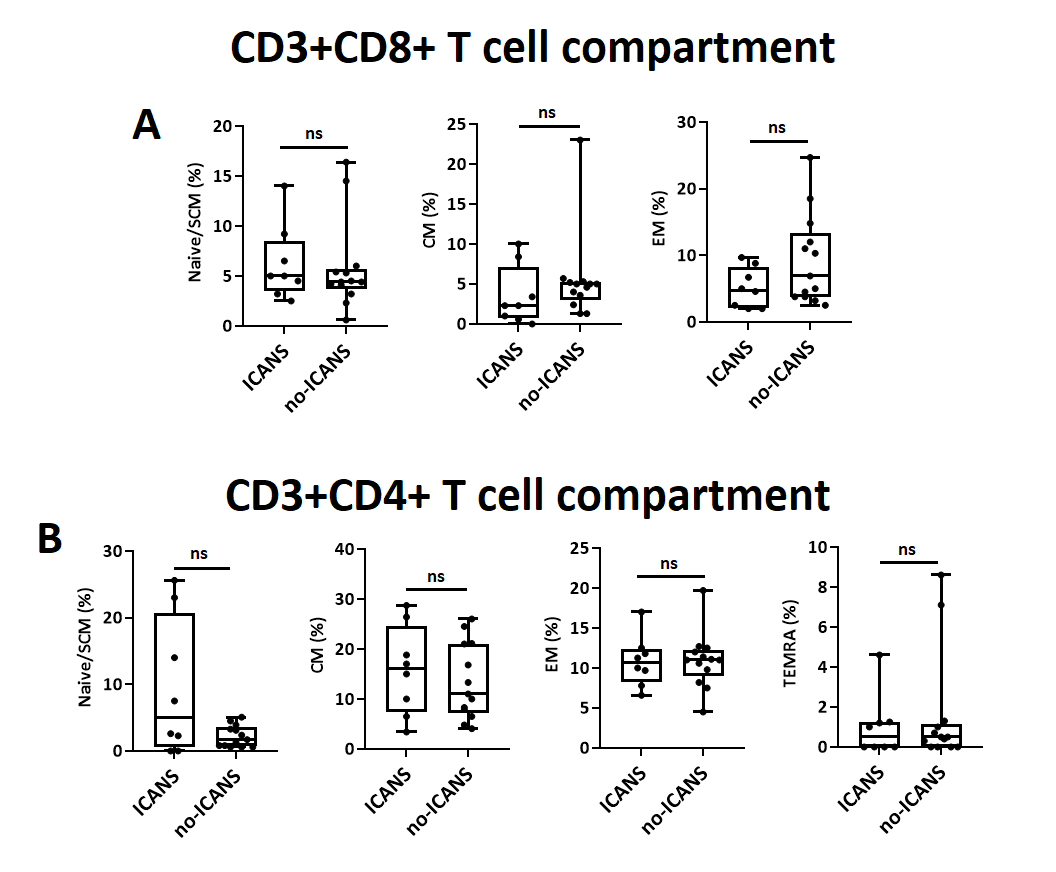

Supplement: Supplementary Figure 1 — Cytofluorimetric analysis. The box plots show the changes in the percentage of Naïve, CM, EM and TEMRA among (A) CD3+CD8+ and (B) CD3+CD4+ T cell compartments at pre-LD in ICANS and no-ICANS patients. Comparisons between 2 groups were made using the non-parametric, unpaired Mann-Whitney test. *p = <0.05; **p = <0.01; ***p = <0.001. [file Image_1.tif]

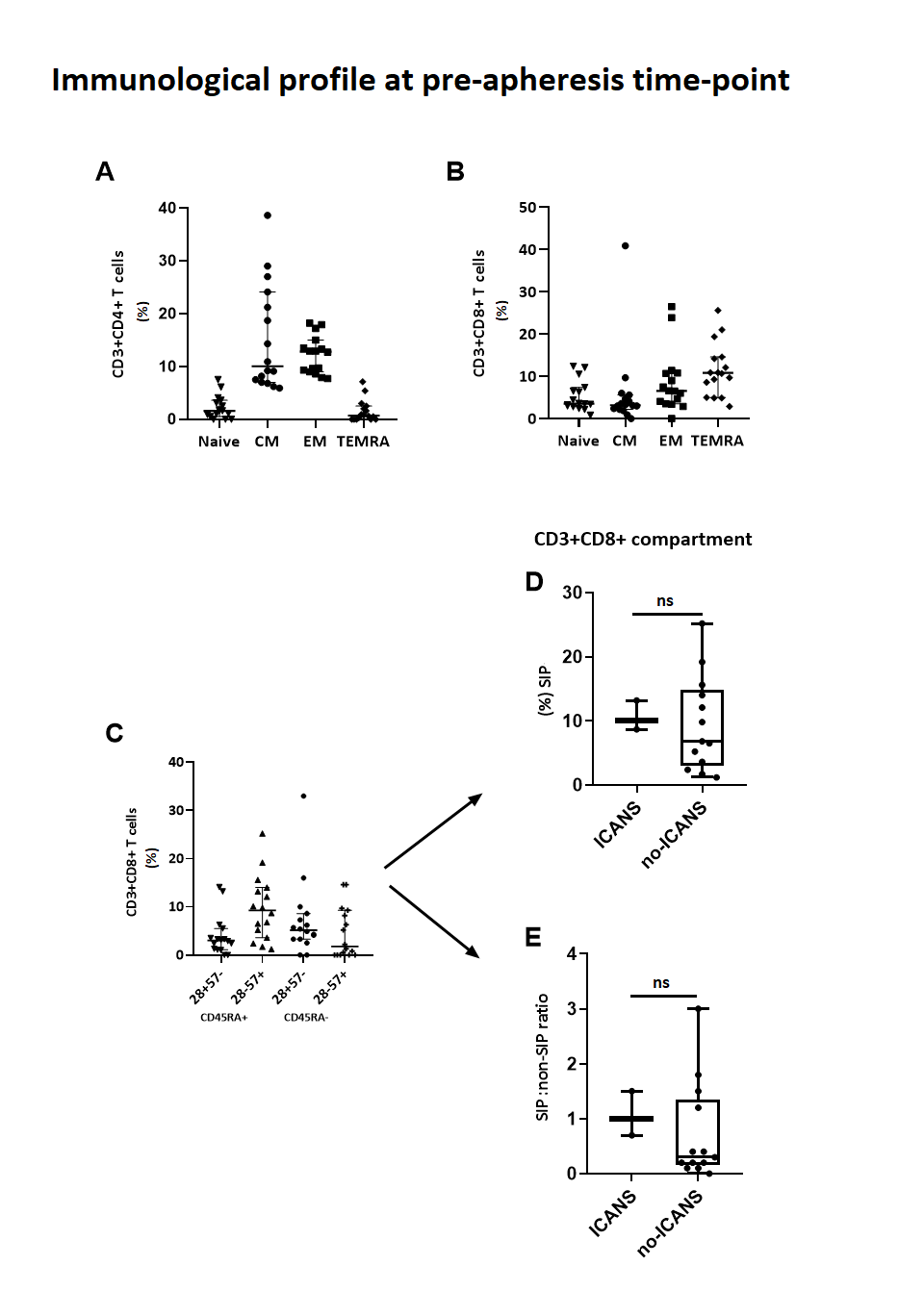

Supplement: Supplementary Figure 2 — Cytofluorimetric analysis. Scatter plots report the distribution of different maturation subsets among (A) CD4, (B) CD8, (C) SIP and non-SIP in CD3+CD8+ T cell compartment at pre-AP; Box plots report the changes in the percentage of SIP (D) and SIP:non-SIP ratio (E) among CD3+CD8+ T cell compartment at pre-AP (F). [file Image_2.tif]

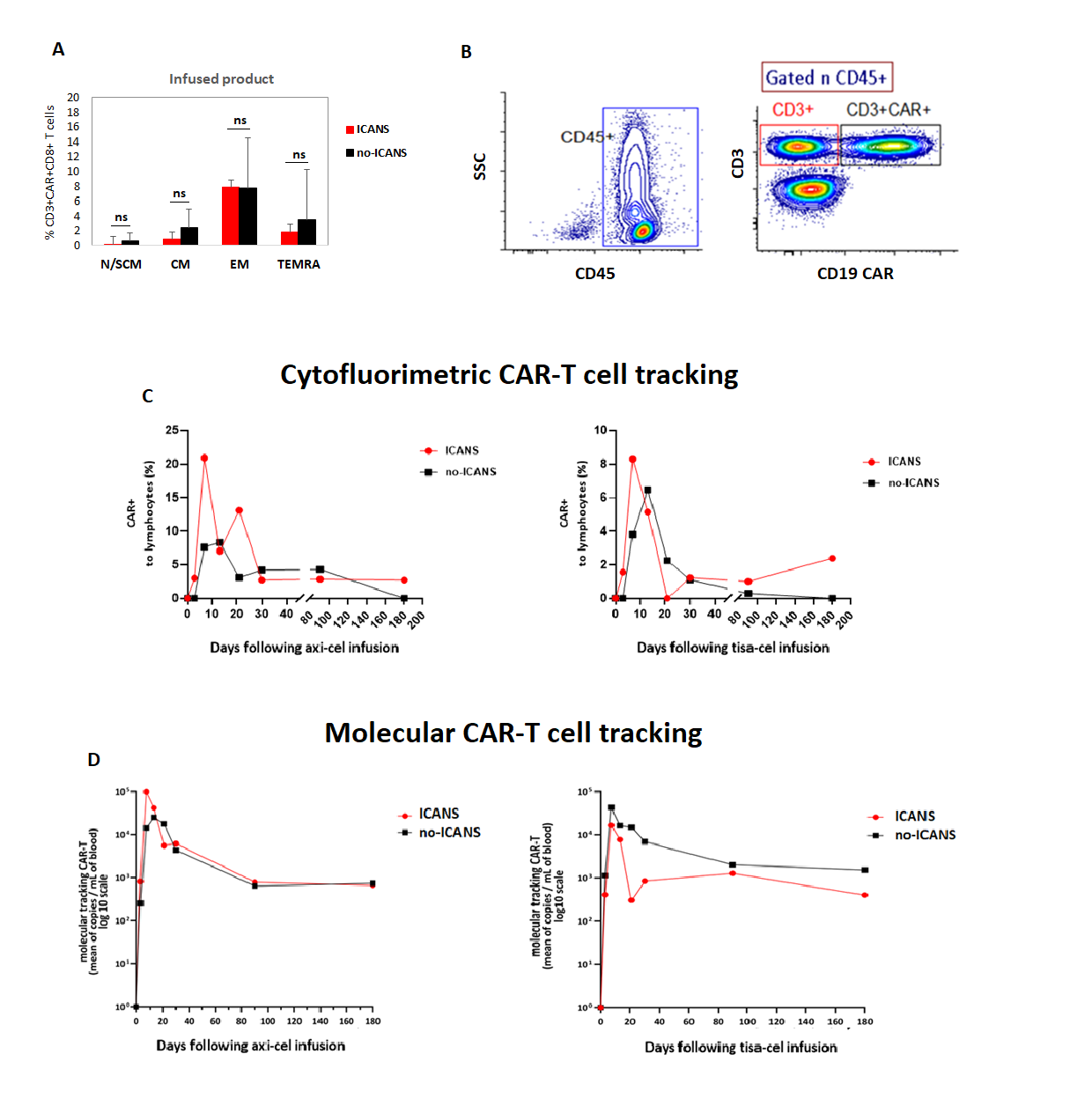

Supplement: Supplementary Figure 3 — Cytofluorimetric analysis. (A) Histograms show the comparison of frequency distribution of Naive, CM, EM and TEMRA cells gated on CD3+CAR+CD8+ cell compartment in infusion product bags leftovers stratified according ICANS. (B) Representative gating strategy of CAR+ T cell population at expansion peak (day+7). CAR-T cell expansion kinetics in patients receiving axi-cel and tisa-cel assessed by (C) flow cytometry and (D) ddPCR (red: patients who developed ICANS; black: no-ICANS patients. Comparisons between 2 groups were made using the non-parametric, unpaired Mann-Whitney test. *p = <0.05; **p = <0.01; ***p = <0.001. [file Image_3.tif]
